# Conversion of percutaneous transhepatic biliary drainage to endoscopic ultrasound‐guided biliary drainage

**DOI:** 10.1002/deo2.6

**Published:** 2021-04-21

**Authors:** Shinichi Morita, Shunsuke Sugawara, Takeshi Suda, Takahiro Hoshi, Satoshi Abe, Kazuyoshi Yagi, Shuji Terai

**Affiliations:** ^1^ Department of Gastroenterology and Hepatology Uonuma Institute of Community Medicine Niigata University Hospital Niigata Japan; ^2^ Department of Diagnostic Radiology National Cancer Center Hospital Tokyo Japan; ^3^ Division of Gastroenterology and Hepatology Graduate School of Medical and Dental Sciences Niigata University Niigata Japan

**Keywords:** adverse event, endoscopic ultrasound‐guided biliary drainage, malignant biliary obstruction, percutaneous transhepatic biliary drainage, recurrent biliary obstruction, re‐intervention

## Abstract

**Introduction:**

Percutaneous transhepatic biliary drainage (PTBD) is a useful alternative treatment for malignant biliary obstruction (MBO) when patients have difficulty with endoscopic transpapillary drainage. We examined the feasibility of conversion of PTBD to endoscopic ultrasound‐guided biliary drainage (EUS‐BD) in patients with MBO unsuited for endoscopic transpapillary biliary drainage.

**Methods:**

This retrospective study included patients who underwent conversion of PTBD to EUS‐BD between March 2017 and December 2019. Eligible patients had unresectable MBO, required palliative biliary drainage, and were not suited for endoscopic transpapillary drainage. Initial PTBD had been performed for acute cholangitis or obstructive jaundice in all patients. EUS‐BD was performed following improvements in cholangitis. Sixteen patients underwent conversion of PTBD to EUS‐BD. We evaluated technical success, procedure time, clinical success (defined as subsequent external catheter removal), adverse events (AEs), time to recurrent biliary obstruction (TRBO), and re‐intervention rates.

**Results:**

Technical success was achieved in all patients (100%). The median procedure time was 45.0 minutes (interquartile range [IQR] 30.0–50.0 minutes). Clinical success was achieved in all patients (100%). There were mild early AEs in two patients (12.5%) (acute cholangitis: 1, bile peritonitis: 1), which improved with antibiotic administration alone. Recurrent biliary obstruction (RBO) occurred in six patients (37.5%). Kaplan‐Meier analysis revealed a 50% TRBO of 95 days (IQR 41–246 days). Endoscopic treatment was possible in all RBO cases, and repeat PTBD was not required.

**Conclusions:**

Conversion of PTBD to EUS‐BD for the management of MBO is both feasible and safe. This approach is expected to be widely practiced at centers with little experience in EUS‐BD.

## INTRODUCTION

Endoscopic transpapillary biliary drainage is the gold standard treatment for malignant biliary obstruction (MBO) before curative and/or palliative cancer treatment.^1^, ^2^, ^3^, ^4^ However, transpapillary biliary drainage can be particularly difficult in certain cases, especially when there is an inability to transverse the biliary stenosis, malignant intestinal obstruction, or surgically altered anatomy.^5^ In such cases, percutaneous transhepatic biliary drainage (PTBD) is performed. Although PTBD is an established alternative treatment for biliary drainage,^6^, ^7^ indwelling external drainage catheters can reduce the patient's quality of life (QOL). Internal stent placement via the PTBD tract for removing the external PTBD catheter is often performed. However, placement may fail if the guidewire cannot be passed through a particularly narrow, long, or tortuous stenosis.^8^ In addition, recurrent biliary obstruction (RBO) due to stent dysfunction necessitates repeat PTBD.

In recent years, endoscopic ultrasound‐guided biliary drainage (EUS‐BD) has been adopted as a secondary method for biliary drainage.^8^, ^9^, ^10^, ^11^, ^12^, ^13^ Recent studies have reported that EUS‐BD achieves therapeutic results equivalent to those of PTBD when endoscopic transpapillary biliary drainage remains difficult.^14^, ^15^, ^16^, ^17^ However, due to its technically challenging nature, the frequency of procedure‐related adverse events (AEs) such as bleeding, abdominal pain, bile leakage, and bile peritonitis remains high.^9^, ^10^, ^18^, ^19^, ^20^

Several studies have reported attempting PTBD before converting the drainage approach to EUS‐BD in an effort to reduce AEs associated with EUS‐BD.^21^, ^22^, ^23^ This approach has several advantages, as initial PTBD can improve the patient's cholangitis. By injecting the contrast medium via the indwelling catheter before EUS‐BD, the bile duct can be expanded and facilitated for puncture under EUS guidance. In addition, the location of the bile ducts can be confirmed through both EUS and fluoroscopy, and the images can be used as a roadmap for advancement of the guidewire.

This single‐center retrospective study aimed to clarify the technical feasibility, efficacy, and safety of conversion of PTBD to EUS‐BD.

## METHODS

This study was approved by the Institutional Human Investigation Committee of the Uonuma Institute of Community Medicine at Niigata University Hospital (30–028). Written informed consent was obtained from all patients prior to procedures in accordance with the tenets of the Declaration of Helsinki.

### Patients

This single‐center retrospective study evaluated the records of patients who underwent conversion of PTBD to EUS‐BD at Uonuma Institute of Community Medicine at Niigata University Hospital in Japan between March 2017 and December 2019. All eligible patients had unresectable MBO, required palliative biliary drainage, and were not suited for endoscopic transpapillary drainage. Patients with bleeding tendencies and massive ascites, which are contraindications for PTBD, were excluded.

**FIGURE 1:**
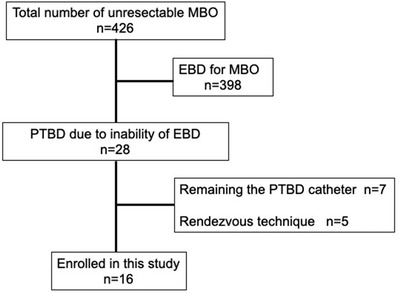
Flow chart depicting enrollment of the study patients Abbreviations: EBD, endoscopic biliary drainage; EUS‐BD, endoscopic ultrasound‐guided biliary drainage; MBO, malignant biliary obstruction; PTBD, percutaneous transhepatic biliary drainage.

Figure 1 shows the flow chart of patient inclusion. PTBD was performed in 28 patients due to an inability to achieve endoscopic transpapillary drainage. Sixteen patients meeting the eligibility criteria were prospectively enrolled.

Conversion of PTBD to EUS‐BD was performed in 16 patients (10 men; median age: 80 years, interquartile range ［IQR: 71–86 years］). The underlying disease was pancreatic cancer in seven patients, hilar bile duct cancer in four patients (Bismuth type 1: 1, type 2: 1, and type 3a: 2), distal bile duct cancer in two patients, ampullary cancer in two patients, and gastric cancer in one patient. The indication for biliary drainage was acute cholangitis in 12 patients and obstructive jaundice in four patients. Endoscopic transpapillary biliary drainage was not possible due to duodenal obstruction subsequent to cancer invasion in 10 patients, surgically altered anatomy in four patients (Roux‐en‐Y reconstruction: 4), and an inability to access the area requiring drainage in two patients. The indwelling branch of the PTBD catheter was B3 of the left lobe in 10 patients and B5 of the right lobe in six patients.

Conversion of PTBD to EUS‐BD was performed following improvements in cholangitis‐associated fever. The median Eastern Cooperative Oncology Group Performance Status score was 1 (IQR: 1–2). The median period from PTBD to EUS‐BD was 9 days (IQR: 6–22 days). The demographic and clinical characteristics of the included patients are summarized in Table 1.

**TABLE 1:** Demographic and clinical characteristics of the 16 included patients

Clinical characteristics	Number of patients (N = 16)
Age (years)	
Median (IQR)	80 (71–86)
Sex, male/female	10/6
Underlying disease	
Pancreatic cancer	7 (43.8)
Hilar bile duct cancer	4 (25.0)
Distal bile duct cancer	2 (12.5)
Ampullary cancer	2 (12.5)
Gastric cancer	1 (6.2)
Indication of initial PTBD, n (%)	
Acute cholangitis	12 (75.0)
Obstructive jaundice	4 (25.0)
Reasons for inability of endoscopic transpapillary drainage	
Duodenal obstruction	10 (62.5)
Surgically altered anatomy	4 (25.0)
Roux‐en‐Y	4
Inability to access the area requiring drainage	2 (12.5)
Bile duct of PTBD catheter placement	
B3 (left lobe)	6 (37.5)
B5 (right lobe)	10 (62.5)
ECOG performance status, median (IQR)	1 (1–2)
Median period from PTBD to EUS‐BD, days (IQR)	9 (6–22)

Abbreviations: ECOG, Eastern Cooperative Oncology Group; EUS‐BD, endoscopic ultrasound‐guided biliary drainage; IQR, interquartile range; PTBD, percutaneous transhepatic biliary drainage.

### PTBD to EUS‐BD conversion technique

All procedures were performed by two gastroenterologists (Shinichi Morita and Satoshi Abe, with 16 and 15 years of experience in gastroenterology, respectively) in the endoscopic‐fluoroscopy suite. Both had extensive experience in PTBD, endoscopic transpapillary drainage, and EUS but had performed an EUS‐BD in fewer than 20 cases. The conversion to EUS‐BD was performed after the initial PTBD and after antibiotic treatment, which sufficiently improved cholangitis and jaundice. Treatment selection for EUS‐hepaticogastrostomy (EUS‐HGS) or EUS‐choledochoduodenostomy (EUS‐CDS) was performed as follows: EUS‐HGS was performed in patients with malignant hilar biliary obstruction, malignant distal biliary obstruction with cancer invasion of the duodenal bulb, and surgically altered anatomy. EUS‐CDS was performed in patients with malignant distal biliary obstruction without cancer invasion of the duodenal bulb, given that it is possible to puncture the bile duct from the duodenum in such cases. Otherwise, EUS‐HGS or EUS‐CDS was performed at the operators’ discretion.

**FIGURE 2:**
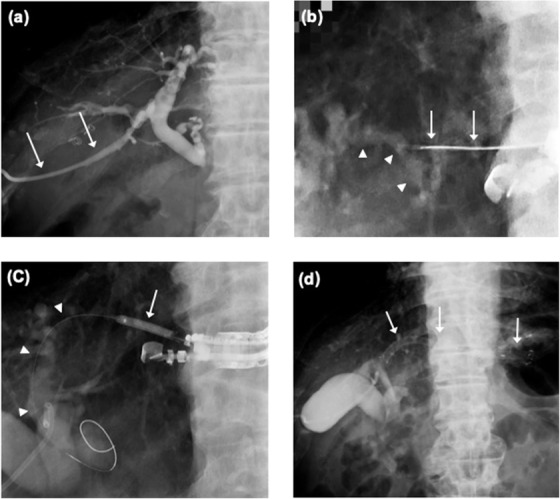
Conversion of PTBD to EUS‐HGS. (a) Cholangiography via the PTBD catheter (arrows). There is no contrast in the distal common bile duct and duodenum due to tumor obstruction of the distal common bile duct. (b) Fluoroscopic image showing the puncture of a 19‐gauge needle (arrows) into the left intrahepatic bile duct (arrow heads). The puncture point can be clearly identified. (c) Fluoroscopic image showing the insertion of a 0.025‐inch guidewire (arrowheads) into the bile duct followed by dilation of the puncture tract with a 4‐mm biliary dilation balloon catheter (arrow). (d) Fluoroscopic image showing placement of a covered metallic stent (arrows) at the appropriate position Abbreviations: EUS‐HGS, endoscopic ultrasound‐guided hepaticogastrostomy; PTBD, percutaneous transhepatic biliary drainage.

A curvilinear echoendoscope (GF‐UCT260; Olympus Medical Systems, Tokyo, Japan) was used. The scope was advanced to the stomach for EUS‐HGS and to the duodenum for EUS‐CDS. Diluted contrast medium was subsequently injected into the bile duct via the indwelling PTBD catheter (Figure 2a), and bile duct expansion was visualized using EUS. A hyperechoic blush was also visualized in the bile duct, and the position of the target bile duct was checked via fluoroscopy. The common bile duct was punctured during EUS‐CDS, while the intrahepatic bile duct of the left lobe was punctured during EUS‐HGS, using a 19‐gauge aspiration needle (Figure 2b). When the indwelling PTBD catheter was placed via the B3 of the left lobe, B2 was selected as the bile duct to be punctured to avoid interference between the puncture needles and the existing PTBD catheter.

After confirming that the target bile duct was properly punctured, the tip of the needle was finely adjusted under fluoroscopic guidance. A sufficient length of 0.025‐inch guidewire was advanced into the bile duct. The puncture tract was dilated over the guidewire using a dilator (ES dilator; ZEON Medical, Tokyo, Japan) followed by dilation with a balloon catheter (4 mm, REN; Kaneka Medics, Osaka, Japan) (Figure 2c). A stent system was advanced coaxially along the guidewire and deployed at the appropriate position (Figure 2d). We used fully covered self‐expanding metallic stents. A 120‐mm HANARO stent with a diameter of 6 mm (Boston Scientific, Natick, MA, USA) was used for EUS‐HGS, while a 60‐mm X‐suit NIR stent with a diameter of 8 mm (Olympus, Japan) was used for EUS‐CDS. After placement of the stent, the contrast medium was injected via the PTBD catheter to confirm stent passage. The PTBD catheter was left in place temporarily and was clamped if no early AEs occurred on the day after EUS‐BD. After the procedure, cefmetazole was administered twice daily (2 g/day). If cholangitis or jaundice did not recur after a few days, indicating successful EUS‐BD, antibiotics were terminated, and the PTBD catheter was removed.

If symptoms of RBO such as high fever, upper abdominal pain, and/or jaundice occurred, re‐intervention was performed if an endoscopic procedure was feasible. Stents that had become almost or completely occluded by sludge accumulation or food impaction were grasped with alligator forceps and carefully removed. After the guidewire was advanced into the fistula, a new metallic stent of equivalent or greater thickness than the original was deployed coaxially over the guidewire.

### Outcome measures

The primary endpoint of this study was technical success, defined as successful placement of a metallic stent in the appropriate position via EUS‐BD, with the assistance of contrast medium injected into the bile duct via the indwelling PTBD catheter. Procedure time, clinical success, AEs, causes of RBO, TRBO, and re‐intervention rates were also evaluated. Clinical success was defined as the ability to appropriately remove the PTBD catheter. All AEs were assessed and graded in accordance with the American Society of Gastrointestinal Endoscopy Lexicon.^24^

Early AEs were those occurring within 2 weeks of stent placement, with subsequent AEs defined as late. RBO was defined as the recurrence of cholangitis and/or jaundice, as evidenced by fever, leukocytosis, increased serum bilirubin level, and biliary dilatation on imaging studies.

All patients were followed up until study termination (March 31, 2020) or death. When patients could not be directly followed up for specific reasons, their family members or personal physicians were contacted by telephone.

### Statistical analysis

Continuous variables are expressed as medians with interquartile ranges, while categorical variables are expressed as numbers and percentages. TRBO, which was evaluated using the Kaplan‐Meier method, was defined as the period from the date of PTBD catheter clamp after EUS‐BD to RBO. Patient death was treated as censored at the time of death. If RBO was not evident before death, TRBO was considered equal to the length of survival. All statistical analyses were performed using GraphPad Prism version 7.0 (GraphPad Software Inc., La Jolla, CA, USA).

## RESULTS

Technical success was achieved in all patients (100%). The median procedure time was 45 minutes (IQR: 30–50 minutes). EUS‐HGS and EUS‐CDS were performed in 14 (87.5%) and two (12.5%) patients, respectively. The median period from the EUS‐BD procedure to the clamping of the PTBD catheter was 5 days (IQR: 2–7 days). Clinical success was achieved in all patients (100%), with removal of the PTBD catheter after a median of 7 days (IQR: 2–9 days). The median length of hospital stay was 14 days (IQR: 8–18 days) (Table 2).

**TABLE 2:** Clinical outcomes and follow‐up data for the 16 included patients

Outcomes	Number of patients (N = 16)
Technical success, n (%)	16 (100)
Median procedure time, minutes (IQR)	45 (30–50)
Replacement of EUS‐BD route, n (%)	
EUS‐HGS	14 (87.5)
EUS‐CDS	2 (12.5)
Clinical success, n (%)	16 (100)
Median period from EUS‐BD to clamping of the PTBD catheter, day (IQR)	5 (2–7)
Median period from EUS‐BD to PTBD catheter removal, days (IQR)	7 (2–9)
Median length of hospital stay, days (IQR)	14 (8–18)
Adverse events, n (%)	
Early adverse events	2 (12.5)
Bile peritonitis	1
Acute cholangitis	1
Recurrent biliary obstruction	6 (37.5)

Abbreviations: EUS‐BD, endoscopic ultrasound‐guided biliary drainage; EUS‐CDS, endoscopic ultrasound‐guided choledocoduodenostomy; EUS‐HGS, endoscopic ultrasound‐guided hepaticogastrostomy; IQR, interquartile range; PTBD, percutaneous transhepatic biliary drainage.

Early AEs occurred in two patients (12.5%), both of which were classified as mild. Acute cholangitis occurred in one patient (6.3%). Within several hours of endoscopic treatment, the patient reported experiencing chills and fever. Antibiotics were administered, and the symptoms improved the next day. Bile peritonitis occurred in one patient (6.3%), who reported abdominal pain with irritation within several hours of the endoscopic procedure. Analgesics and antibiotics were administered, and the patient's abdominal pain disappeared the next day.

**FIGURE 3:**
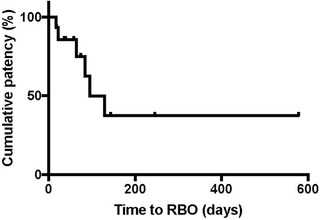
Kaplan‐Meier graph showing TRBO after successful conversion of PTBD to EUS‐BD. The 50% TRBO was 95 days (IQR: 41–246 days) Abbreviations: EUS‐BD, endoscopic ultrasound‐guided biliary drainage; IQR, interquartile range; PTBD, percutaneous transhepatic biliary drainage; TRBO, time to recurrent biliary obstruction.

RBO occurred in six patients (37.5%) (Table 3). The cause of RBO was sludge accumulation in four patients, food impaction in one patient, and distal stent migration in one patient. Kaplan‐Meier analysis revealed a 50% TRBO of 95 days (IQR: 41–246 days) (Figure 3). Sludge accumulation occurred on the 64th, 84th, 95th, and 129th days after PTBD catheter clamp, all cases of which occurred after EUS‐HGS. Stent exchange was performed in all cases. The patient with food impaction presented with cholangitis and septic shock on the 22nd day after PTBD catheter clamp followed by EUS‐CDS. Given the patient's unstable general condition, an endoscopic nasobiliary drainage catheter was first placed via the choledocoduodenal stent into the intra‐hepatic bile duct. After the patient's general condition improved, the existing stent was exchanged with a thicker stent (10 mm in diameter). The case of stent migration was discovered on computed tomography performed subsequent to a high fever that appeared on the 15th day after PTBD catheter clamp followed by EUS‐HGS. Endoscopy confirmed that the existing fistula remained in the shape of a pinhole. A guidewire was passed through the fistula into the bile duct, and a similar stent was placed using the over‐the‐guidewire technique.

**TABLE 3:** Details of cases of recurrent biliary obstruction

	Drainage method	Type of stent	Cause of RBO	Period from the date of PTBD catheter clamp to RBO (days)	Treatment
Case 1	EUS‐HGS	FCSEMS, 6 × 120 mm	Sludge	84	Stent exchange
Case 2	EUS‐HGS	FCSEMS, 6 × 120 mm	Sludge	95	Stent exchange
Case 3	EUS‐HGS	FCSEMS, 6 × 120 mm	Stent migration	15	Stent re‐placement
Case 4	EUS‐CDS	FCSEMS, 8 × 80 mm	Food impaction	22	ENBD, stent exchange
Case 5	EUS‐HGS	FCSEMS, 6 × 120 mm	Sludge	129	Stent exchange
Case 6	EUS‐HGS	FCSEMS, 6 × 120 mm	Sludge	64	Stent exchange

Abbreviations: ENBD, endoscopic nasobiliary drainage; EUS‐CDS, endoscopic ultrasound‐guided choledocoduodenostomy; EUS‐HGS, endoscopic ultrasound‐guided hepaticogastrostomy; FCSEMS, fully covered self‐expandable metallic stent; PTBD, percutaneous transhepatic biliary drainage; RBO, recurrent biliary obstruction.

During a median follow‐up period of 134 days (IQR: 71–272 days), 11 patients (68.8%) died due to underlying disease, and five survived to the end of the study.

## DISCUSSION

In the present study, all EUS‐BD procedures were successful without serious treatment‐related AEs, despite both operators having previously performed fewer than 20 EUS‐BD procedures. Our findings indicated that it was possible to perform EUS‐BD in either the left or right intrahepatic bile duct in which the initial PTBD catheter was placed. Moreover, removal of the external PTBD catheter was possible in all patients. Although RBO occurred in six patients during the study period, endoscopic treatment was possible in all cases, and repeat PTBD was not required.

There are several clinical advantages to conversion of PTBD to EUS‐BD. For patients with poor general or respiratory status, such as those with septic shock subsequent to acute cholangitis, initial PTBD helps to improve their general condition and allows for subsequent endoscopic treatments that require sedation. Shrinkage of the bile duct after PTBD makes its puncture difficult; however, injection of saline or contrast medium via the catheter can re‐expand the bile duct and facilitate puncture under EUS guidance. Contrast injection and bile duct cholangiography can also be used as a roadmap to guide the procedure, assist with puncture, advance the guidewire into the bile ducts, and subsequently reduce the risk of entry into undesired locations, such as the portal vein. If an AE occurs, the initial PTBD would have already secured a bile drainage route, which can be used to minimize bile leakage into the abdominal cavity. Therefore, this approach may be useful for the operators, as it simplifies the EUS‐BD procedure, and the initial PTBD can act as a salvage drainage route if AEs occur.

PTBD for MBO is a long‐established treatment that remains widely utilized in patients who are unable to undergo endoscopic transpapillary biliary drainage.^6^, ^7^ However, a major disadvantage of this technique is the remnant indwelling catheter, which impairs patients’ QOL. In recent years, EUS‐BD has been gradually adopted as an alternative biliary drainage method for patients in whom transpapillary biliary drainage is not possible.^8^, ^9^, ^10^, ^11^, ^12^, ^13^ Moreover, some studies have reported that the treatment results of EUS‐BD are equivalent to those of PTBD.^14^, ^15^, ^16^, ^17^ Furthermore, researchers have reported that EUS‐BD is superior when comparing endoscopic retrograde cholangiopancreatography (ERCP) and EUS‐BD for the initial treatment of MBO. Given these findings, the number of patients undergoing EUS‐BD for MBO is expected to increase.^25^

However, EUS‐BD is technically difficult and remains associated with a high AE rate, ranging from 16.5% to 23.3%,^9^, ^10^, ^11^, ^12^, ^18^, ^19^, ^20^ which is higher than that for ERCP and PTBD. Furthermore, rates of successful treatment are low in the hands of inexperienced operators, and several fatal AEs have been reported.^18^, ^19^, ^20^ Therefore, EUS‐BD is mainly performed at high‐volume centers, and it is assumed that the AE rate is higher in low‐volume general hospitals. EUS‐BD remains a newer procedure that is less widely adopted than PTBD. However, a previous study reported that the technical success rate, stent patency, and AE rate of EUS‐BD are superior to those of other drainage treatments for operators with sufficient EUS‐BD experience.^25^ Therefore, the treatment is considered useful following the initial technical learning curve.^20^ Thus, selection of PTBD or EUS‐BD should be based on the clinical situation and the skill level of the operator.

Several studies have reported initial PTBD before conversion to EUS‐BD in an effort to reduce the AE rate.^21^, ^22^, ^23^ Paik et al retrospectively examined the conversion of PTBD to EUS‐BD in 16 patients treated at two high‐volume centers in whom internal biliary stent via the PTBD tract was not possible.^23^ The technical success rate was 100%, and no serious treatment‐related AEs were observed. While this retrospective study was performed in two high‐volume centers, we observed similar results in our study even at centers with little experience in EUS‐BD. Two patients in our study developed mild early AEs after EUS‐BD (bile peritonitis and acute cholangitis, respectively). We believe that repeated cholangiography via the PTBD catheter during the procedure caused retrograde infection and bile leakage from the punctured fistula into the abdominal cavity prior to the placement of the fully covered self‐expandable metal stent. In addition, overexpansion of the fistula may increase the risk of bile leakage into the abdominal cavity. In order to avoid bile leakage, further technological improvements may be needed to reduce the number of device exchanges during the procedure.^26^, ^27^, ^28^ Furthermore, some studies have reported gallbladder drainage during the conversion from percutaneous drainage to internal drainage under EUS guidance.^29^ As observed in the present study, the authors reported high technical and clinical success rates for this technique. However, in the previous study, the indwelling percutaneous catheter may have induced thickening and stiffness of the gallbladder wall, potentially hindering endoscopic puncture and dilatation of the puncture tract. In contrast, we observed no cases in which endoscopic puncture was difficult due to thickening of the bile duct wall, although the possibility of this issue must be considered.

This study has several limitations, including its retrospective design and small sample size. Second, we did not compare outcomes with those observed following an initial EUS‐BD strategy. Considering the AE rate of PTBD, performing both PTBD and EUS‐BD may increase the overall risk of AEs. PTBD has been associated with several AEs,^16^, ^17^, ^30^ and it is important to confirm whether the patient's general condition and antiplatelet and anticoagulant regimen are appropriate. It is also necessary to consider the reduction in QOL associated with PTBD catheter placement. Furthermore, stent placement via the PTBD tract (PTBDS) was not performed in this study. PTBDS of the tumor stenosis is the current standard technique and is often performed to internalize and remove the external PTBD catheter. However, PTBDS can be difficult if the duodenum contains a malignant obstruction or if guidewire passage through the stenosis is difficult due to a tough, long, or tortuous bile duct.^23^ The success rate of PTBDS treatment ranges from 46% to 93%, with an AE rate of 43%.^8^, ^31^ In addition, when RBO occurs due to a stent dysfunction, the endoscopic transpapillary approach is difficult in patients for whom the initial ERCP was challenging as well, which would necessitate a repeat PTBD. The progress made in multidisciplinary cancer treatment in recent years has gradually improved patient prognosis while also increasing the frequency of re‐interventions. Therefore, our approach is useful because it enables endoscopic re‐intervention through a previously created transmural drainage route. In this study, although six patients experienced RBO requiring re‐intervention, endoscopic stent exchange via the EUS‐BD fistula was possible in all cases.

In conclusion, our findings demonstrate that conversion of PTBD to EUS‐BD in patients with an unresectable MBO in whom an endoscopic transpapillary approach would be difficult is both technically feasible and relatively safe. Further investigations are required to evaluate the efficacy of conversion from PTBD to EUS‐BD.

## CONFLICT OF INTEREST

The authors declare that there is no conflict of interest that could be perceived as prejudicing the impartiality of the research reported.

## FUNDING INFORMATION

None.
